# Insulin reference intervals in Brazilian adolescents by direct and indirect approaches: validation of a data mining method from laboratory data

**DOI:** 10.1016/j.jped.2024.03.009

**Published:** 2024-04-23

**Authors:** Monica D.C. Freire, Paulo R.T.P. Dias, Thiago S.P. Souza, Caio K. Hirose, Paula B.M.C. Araujo, Mario F.T. Neves

**Affiliations:** a Universidade do Estado do Rio de Janeiro, Pós Graduação em Ciências Médicas, Rio de Janeiro, RJ, Brazil; b Universidade Federal Fluminense, Instituto de Saúde Coletiva, Departamento de Epidemiologia e Bioestatística, Niterói, RJ, Brazil; c Instituto de Ensino e Pesquisa DASA, São Paulo, SP, Brazil; d Universidade do Estado do Rio de Janeiro, Núcleo de Estudos e Pesquisas em Atenção ao Uso de Drogas, Rio de Janeiro, RJ, Brazil; e Universidade do Estado do Rio de Janeiro, Instituto de Matemática e Estatística, Rio de Janeiro, RJ, Brazil; f DASA, Analytics Department, São Paulo, SP, Brazil; g Universidade Federal do Rio de Janeiro, Faculdade de Medicina, Pós-graduação em Endocrinologia, Rio de Janeiro, RJ, Brazil; h Universidade do Estado do Rio de Janeiro, Faculdade de Ciências Médicas, Rio de Janeiro, RJ, Brazil

**Keywords:** Data mining, Insulin, Reference interval, Adolescent

## Abstract

**Objective:**

To determine reference intervals (RI) for fasting blood insulin (FBI) in Brazilian adolescents, 12 to 17 years old, by direct and indirect approaches, and to validate indirectly determined RI.

**Methods:**

Two databases were used for RI determination. Database 1 (DB1), used to obtain RI through *a posteriori* direct method, consisted of prospectively selected healthy individuals. Database 2 (DB2) was retrospectively mined from an outpatient laboratory information system (LIS) used for the indirect method (Bhattacharya method).

**Results:**

From DB1, 29345 individuals were enrolled (57.65 % female) and seven age ranges and sex partitions were statistically determined according to mean FBI values: females: 12 and 13 years-old, 14 years-old, 15 years-old, 16 and 17 years-old; and males: 12, 13 and 14 years-old, 15 years-old, 16 and 17 years-old. From DB2, 5465 adolescents (67.5 % female) were selected and grouped according to DB1 partitions. The mean FBI level was significantly higher in DB2, on all groups. The RI upper limit (URL) determined by Bhattacharya method was slightly lower than the 90 % CI URL directly obtained on DB1, except for group female 12 and 13 years old. High agreement rates for diagnosing elevated FBI in all groups on DB1 validated indirect RI presented.

**Conclusion:**

The present study demonstrates that Bhattacharya indirect method to determine FBI RI in adolescents can overcome some of the difficulties and challenges of the direct approach.

## Introduction

Reference intervals (RI) for laboratory tests are fundamental tools in clinical practice as they provide a critical framework for assessing and interpreting laboratory results. By comparing a patient's values to these predefined intervals, healthcare professionals can detect abnormalities, diagnose diseases, and make informed decisions regarding treatment and patient care. Hence, appropriately defined reference values are of vital importance.^1^

Every laboratory should establish RI suitable for its specific service populations, according to the International Federation of Clinical Chemistry (IFCC) Clinical Laboratory Standards Institute (CLSI), since RI might be affected by race, age, sex, region, and other factors.^2^ This partitioning is of utmost importance in pediatrics, as physiological advances after birth and during adolescence result in changes in many laboratory analytes.^3^ Therefore, the use of wide age groups or missing sex grouping in adolescence can be intrinsically associated with inaccuracy.^4^

The determination of RI can be done through direct and indirect methods.^2^ IFCC, to this day, considers the *a priori* direct method as the gold standard, but it has considerable drawbacks, such as difficulty in recruiting sufficient reference individuals; selection bias in small-sample data; and time, labor, and financial costs.^5^ In addition, for special groups, such as in pediatric population, it is difficult to obtain a large number of apparently healthy individuals. Transference of RI from different populations – an alternative to the determination of one's laboratory own RI - may result in a marked variation of sample classification, affecting diagnosis and treatment.^6^

As an alternative to clinical studies, the indirect approach, determines RI from data mined from existing laboratory datasets by calculation through various mathematical methods available.^7^ Indirect methods may be more feasible to determine RI specific to local populations, especially appropriate in vulnerable populations, where ethical considerations and practical problems often hinder the recruiting of sufficient numbers of healthy volunteers.

With the advent of medical big data, the use of indirect methods based on data sets previously obtained from clinical laboratories is promising. The establishment of RI using indirect methods roughly involves data acquisition, data cleaning, transformation of skewed data, elimination of outliers or error values, and selection of appropriate statistical methods to calculate the reference limits (RLs).^8^

Nowadays, childhood obesity is an emerging problem.^9^ The rising prevalence of obesity is causing an increase in obesity-related complications such as insulin resistance.^10^ Hyperinsulinemia is a normal physiological state during puberty,^11^ but children with obesity can have abnormally high fasting blood insulin (FBI) levels.^12^

The gold standard for the assessment of IR is the hyperinsulinemic euglycemic clamp, which is invasive, expensive, and complex to use in the daily clinical practice.^11^ Therefore, the most common surrogate markers of IR are the fasting plasma insulin and the homeostasis model assessment insulin resistance (HOMA-IR).^10^

The aim of this study was to determine RI for FBI in Brazilian adolescents - by *a posteriori* direct approach using the ERICA Study database^13^ and by an indirect approach, using LIS outpatient data mining and Bhattacharya method – and, by compare their results, evaluate the data mining indirectly determined RI.

## Materials and methods

### *Study design and subjects*

The study determined RI for FBI from 2 different databases of Brazilian adolescents, from 12 years-old to 17 years, 11 months, and 30 days old (from now called 17 years-old), according to age and sex partitions. DB1 consists of a reference population of healthy individuals, from 124 Brazilian cities, recruited from January 2013 to November 2014,^2^ as part of a large population study on cardiovascular risk in adolescence, the ERICA study.^13^ DB2 was obtained by data mining the laboratory information system (LIS) of a large commercial laboratory, from outpatients whose samples were directly drawn by the laboratory, from four States and the Federal District of Brazil, from January 2011 to June 2015.

Exclusion criteria for DB1 were fasting glucose equal or higher than 126 mg/dL, glycated hemoglobin equal or higher than 6.5%, self-reported pregnancy or suspicion of pregnancy, self-reported acute or chronic illness, and regular prescription drug use. DB2 included individuals only once,^14^ based on the first FBI determination observed on the database timeline, and excluded females with a positive BHCG testing, anytime 9 months prior to the FBI test result observed.

Outliers were removed from both data sets prior to partitioning, after data log transformation, by the Chauvenet criteria.

Age and sex partitions were calculated on DB1 and used as partitions on DB2 as well.

The authors used the RefVal software to perform *a posteriori* direct method calculation of RI for FBI for DB1^2^, and Bhattacharya indirect method obtain RI from DB2.^15^^,^^16^

Based on the direct and indirect RI obtained, the authors evaluated the rates of agreement of RI upper limits (URL) in diagnosing elevated FBI levels on individuals in DB1.

FBI analyses were performed by the same commercial laboratory on both databases, on 5 laboratory sites throughout Brazil using standard operations, on Roche Diagnostics electrochemiluminescence platform. The laboratory is accredited by the Clinical Laboratories Accreditation Program of the Brazilian Society of Clinical Pathology and Laboratory Medicine. Standard pre-analytical methods and materials were determined and instructed by the same laboratory for the collection and sample handling on both databases.

This study was approved by the Ethics Committee of the Pedro Ernesto University Hospital – Universidade do Estado do Rio de Janeiro, under the document number: 2.970.023. Data were analyzed using R software 4.2.3, RefVal software and Microsoft Excel - Office 365.

FBI levels and RI were presented in µIU/mL. RI represents the values between the lower and upper reference limits. Standard deviations are also available per partition.

### Age and sex partitions

After data log-transformation, initial age and sex partitions were defined arbitrarily using DB1: males 12, 13, 14, 15, 16, and 17 years-old, and female 12, 13, 14, 15, 16, and 17 years-old. Then the authors used the Bartlett test to approach the homogeneity of variances for each group (transformed data). Since variance results were not homogeneous, the authors used the Kruskal-Wallis test to evaluate if there were significant differences in FBI DB1 measurements on age and sex groups. As they were present, the authors performed multiple pairwise paired *t*-tests between the levels of the within-subject groups. P-values are adjusted using the Bonferroni multiple-testing correction method. Groups with no statistically significant differences were merged into a single partition.

The authors then applied the DB1 partitions, considered the reference sample obtained by individual interview and physical examination for the gold standard RI calculation method, at DB1, once DB1 is the data set with the highest percentage of “healthy” subjects. This allowed comparisons between DB1 and DB2 RI, per partition.

### Direct approach RI

DB1 was used for the determination of RI by the *a posteriori* direct method, for each age and sex group, as described by IFCC C28-A3c Guidelines.^2^ The authors performed RI direct estimates of log-transformed FBI levels, after outlier exclusion, per partition, by parametric and bootstrap methods. RI represents the central 95 % range. Lower and upper reference limits correspond to the 2.5th and 97.5th percentiles, respectively. The authors additionally determined their 90 % confidence intervals (90 % CI).

### Indirect approach RI

After data log transformation, subsequent outlier removal and partitioning, DB2 was used for the indirect determination of RI, for each age and sex partition, by a mathematical mixture separation method, the Bhattacharya method, widely used and described on literature.^15^ In brief, a graphical method is used to identify an underlying Gaussian distribution amongst other data, which may include an “unhealthy” dataset of individuals.

### *Validation of the indirectly determined fasting blood insulin URL*

#### Comparison of the diagnosis of elevated FBI of DB1 individuals

The authors identified the diagnosis of elevated FBI by the indirectly determined URL on every subject of DB1 and compared it to the diagnosis obtained when using the directly determined 90 % CI URL. The question addressed is if a subject's FBI measurement would have different clinical interpretations when compared to the highest probabilistic gold-standard URL - in this case, the higher limit of 90 % CI of the direct URL- and when compared to the indirectly determined URL.

Four groups of results were determined according to the diagnostic agreement, for sex and age partitions: FBI below DB1 90 % CI URL and DB2 URL; B: FBI above DB1 90 % CI URL and below DB2 URL; C: FBI below DB1 90 % CI URL and above DB2 URL; D: FBI above DB1 90 % CI URL and DB2 URL. This analysis was used to evaluate the consistency of the decision impact caused by the indirectly determined URL, compared to the directly determined URL.

## Results

### *Database population distribution and FBI level results*

DB1, derived from the ERICA Study, consisted of 29,200 individuals from 12 to 17 years old, after exclusion criteria application. Outliers removed represented 1.2 % (453) of the sample, therefore 28,747 individuals were selected for partitioning and subsequent RI determination. Most of the participants were female (*n* = 16,919; 58.85 %). Population distribution after removal of outliers, and before partitioning was not normal. Age and sex groups determined were: female 12 and 13 years-old (*n* = 4775); female 14 years-old (*n* = 3035); female 15 years-old (*n* = 3164); female 16 and 17 years-old (*n* = 5945); male 12 to 14 years-old (*n* = 5451); male 15 years-old (*n* = 2315); and, male 16 and 17 years-old (*n* = 4062).

DB2 consisted of 5895 adolescents, after exclusion criteria. The distribution prior to the removal of outliers and partitioning was not normal. After log-transformation and removal of outliers (*n* = 532; 8.27 %), 3692 were female (62.6 %). Then, age and sex partitions from DB1 were used to group DB2 subjects.

The mean FBI measurements – recalculated from the log-transformed results - were significantly higher in DB2, on all groups. Table 1 shows the distribution characteristics and FBI measurements of both databases.

**Table 1:** Age and sex partitions, number of subjects and FBI central measures.

	data base 1			data base 2			p value
	n	FBI mean (µIU/mL)	SD	n	FBI mean (µIU/mL)	SD	
Females							
12–13 yo	4775	10.2	4.85	928	15.8	8.23	<0.001
14 yo	3035	9.45	4.68	478	13.6	7.48	<0.001
15 yo	3164	8.97	4.46	532	13.9	8.20	<0.001
16–17 yo	5945	8.59	4.35	1754	12.8	7.78	<0.001
Males							
12–14 yo	5451	8.43	4.76	1187	14.0	8.28	<0.001
15 yo	2315	7.98	4.51	320	13.1	8.53	<0.001
16–17 yo	4062	7.33	4.32	696	12.1	8.21	<0.001

FBI, fasting blood insulin.

### *Reference intervals for FBI in adolescents*

**Figure 1:**
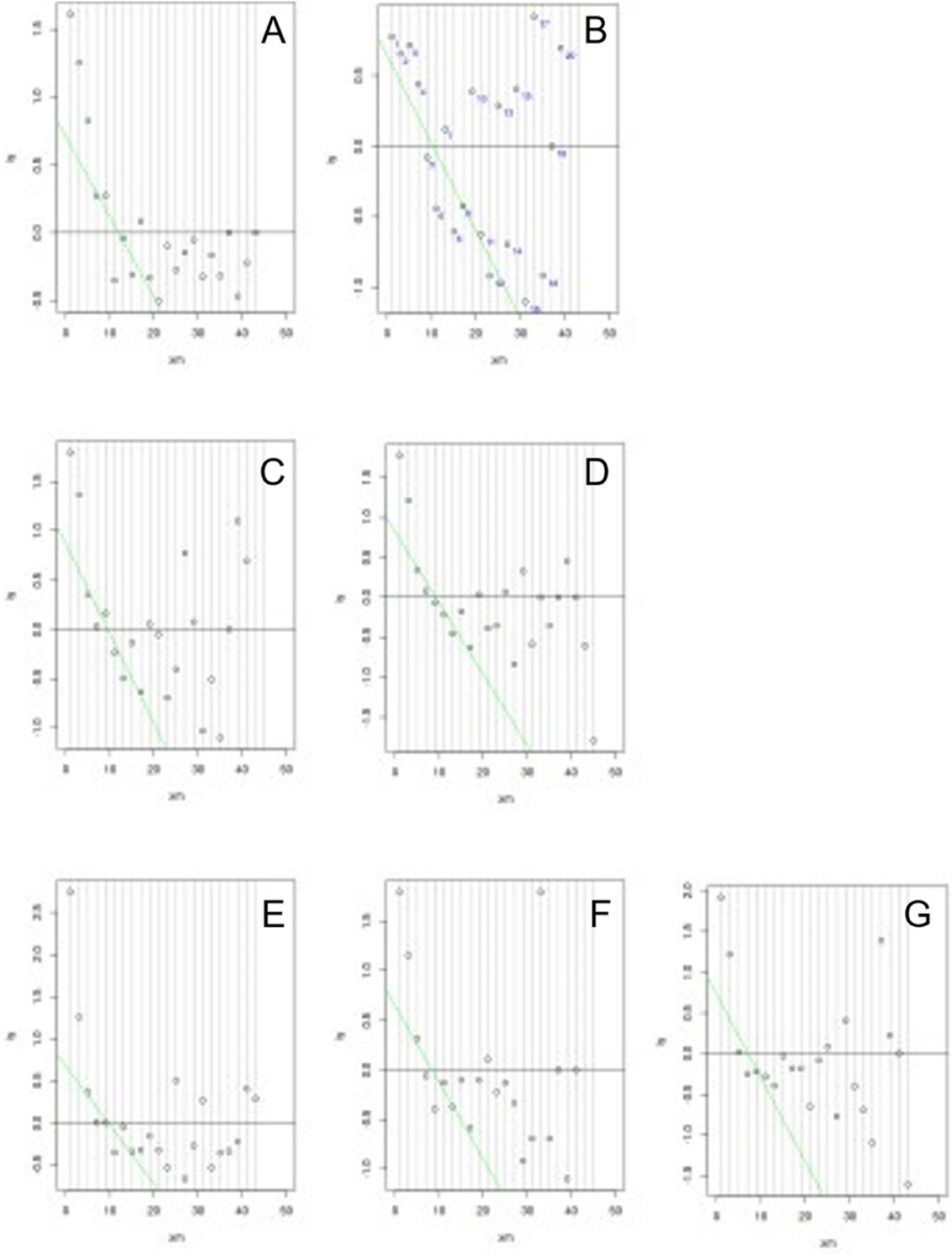
Plot of the Bhattacharya method applied to data-mined LIS data base (DB2), per age and sex partition. A, Female 12 and 13 years-old; B, Female 14 years-old; C, Female 15 years-old; D, Female 16 and 17 years-old; E, Male 12 to 14 years-old; F, Male 15 years-old; G, Male 16 and 17 years-old. Linear region represents the distribution of “healthy” individuals and RI are determined by the extension of the linear region of the “healthy” group.

URL determined by Bhattacharya method (Figure 1) were slightly lower than the 90 % CI URL directly obtained on DB1, except for group female 12 and 13 years-old. (24.27 µIU/mL versus 22.9 µIU/mL). Table 2 shows RI determined per age and sex group, by both methods.

**Table 2:** Reference intervals established by *a posteriori* direct method and indirect method (Bhattacharya method).

	Data base 1 (Direct)				Data base 2 (Bhattacharya)	
	LRL	LRL 90 %CI	URL	URL 90 %CI	LRL	URL
Females						
12–13 years-old	2.6	2.4–2.7	22.2	21.7–22.9	1.6	24.27
14 years-old	2.2	1.9–2.5	21.4	20.6–22.9	0.2	22.33
15 years-old	2.3	2.1–2.5	20.9	19.9–21.5	1.36	19.62
16–17 years-old	2.1	1.9–2.3	19.5	19.1–20.1	0.96	19.38
Males						
12–14 years-old	2	1.9–2.1	20.8	20.3–21.3	0.18	21.02
15 years-old	1.88	1.5–2.0	20.2	19.9–21	0.57	19.24
16–17 years-old	1.6	1.4–1.7	19.3	18.6–20.2	0.5	16.62

LRL, lower reference limit; URL, upper reference limit; LRL 90 %CI, lower reference limit 90 % confidence interval; URL 90 %CI, upper reference limit 90 % confidence interval; FBI, fasting blood insulin.

### *Diagnostic agreement of elevated FBI between direct and indirect URL*

The directly determined 90 % CI URL, and the URL calculated by the Bhattacharya method were used to assess if the FBI level of each DB1 individual subject was considered elevated. The authors observed high agreement rates for elevated FBI levels, in all partitions (Table 3).

**Table 3:** Diagnostic agreement rates for elevated FBI per age and sex group, at DB1.

	A	B	C	D		diagnostic agreement (%)
Females						
12–13 years-old	4675	34	0	66		99.29 %
14 years-old	2972	0	2	61		99.93 %
15 years-old	3068	0	33	63		98.96 %
16–17 years-old	5787	0	29	129		99.51 %
Males						
12–14 years-old	5321	0	13	117		99.76 %
15 years-old	2236	0	33	46		98.57 %
16–17 years-old	3880	0	99	83		97.56 %

A, FBI below DB1 URL (90 % CI) and DB2 URL; B, FBI above DB1 URL (90 % CI) and below DB2 URL; C, FBI below DB1 URL (90 % CI) and above DB2 URL; D, FBI above DB1 URL (90 % CI) and DB2 URL.

## Discussion

Fasting blood insulin level is an important biomarker for insulin resistance observed in overweight, obesity and metabolic syndrome, conditions related to cardiovascular risk.^17^ Healthy adolescents respond to normal metabolic changes with increased insulin resistance. Therefore, FBI levels physiologically also increase in pubertal youngsters.^11^ The present findings on the FBI RI in different groups of adolescents, regardless of the methodology used, corroborate this physiological knowledge. A systematic review conducted by Van der Aa et al. showed insulin resistance prevalence rates from 3.1% to 44%.^10^ The authors used six different methods to define insulin resistance and a variety of cut-offs, leaving a gap of consensus regarding RI for FBI in pediatrics. This highlights the importance of understanding the URL of blood insulin levels in adolescents to properly address pathological insulin resistance in this population.

In recent years, several studies on pediatric RI of multiple analytes using both direct and indirect approaches have been published.^18^, ^19^, ^20^, ^21^, ^22^ Large multicentric studies attempted to determine pediatric RI to several analytes. CALIPER Study by Colantonio et al. presented fasting insulin results from 190 individuals between 6 and under 19 years old, but not its RI.^22^ Kelly et al. published *a posteriori* direct calculated RI for insulin from 240 subjects from 11 to 19 years-old, without further age or sex partitions, and observed extremely high URL obtained by Abbott Architect platform.^23^ Later, Adeli et al. in a collaboration between The Canadian Health Measures Survey and CALIPER initiative, were able to use insulin measurements processed on Centaur XP and Immulite 2000 platforms from 1700 adolescents from 11 to 19 years-old and proposed the same lower (2.1 µIU/mL) and upper (21.8 µIU/mL) limits for both boys and girls without additional age partitions.^4^ However, age and sex partitions are key to pubertal adolescents since insulin resistance varies according to Tanner stages in males and females.^11^ The present study, on the other hand, brings the novelty of establishing insulin RI addressing this issue.

It is known that uncertainties and differences between a reference population-derived RI and the local population served by a specific laboratory can affect the diagnostic accuracy if the laboratory simply verifies or transfers these RI to apply in other populations than the original one.^24^ Additionally, accurate determination of RI, directly or indirectly calculated, and comparison between methods, can be affected by biological variables that influence and demand appropriate group separation (homogeneity, age, sex, nutritional state, ethnicity).^25^^,^^26^ Both RI estimation methods can lead to erroneous RI if stratification for variables is not performed properly. Despite the Brazilian geographical broadness, DB1 and DB2 samples in this study were analyzed on five sites of the same commercial laboratory, by the same analytical platform (Roche Diagnostics) in order to optimize quality control and the use of similar standard operational procedures. Also, since the unknown fraction of the pathological distribution can affect the capability of separation of groups from DB2, the authors calculated age and sex partitions from DB1, which enrolled only healthy subjects after individual interview and examination, and then applied them to DB2 calculations.^25^ The large number of individuals enrolled in the ERICA Study not only allowed statistically calculated DB1 age and sex partitions but provided the highest number of adolescents for the direct calculation of FBI RI in the literature.^13^

Mathematical indirect methods, such as Bhattacharya and others, can be calculated without the strict need to identify and exclude individuals according to signs of disease or to prefer individuals with signs of “good health”. It uses mixed data sets that are mainly composed of healthy persons, with some percentage of “diseased” ones (less than 20%).^8^ The authors observed that mean FBI measurements were significantly higher on DB2 than DB1, as it represents the expected mixture of “healthy” and “unhealthy” subjects. Still, efforts to exclude known potential “non-healthy” individuals can improve the quality of the indirect RI obtained and can be achieved by exclusion criteria, such as: eliminating inpatients and pregnant women; using selected health records such as clinical history or laboratory findings; eliminating subjects with more than one analyte measurement as an indication of ongoing disease investigation; and by removal of outliers.^14^ However, filtering algorithms may not be enough to accurately determine indirect RI.^25^ Mathematical methods should be added to further separate and identify the subjacent unknown “non-diseased” population. With the development of informatics technology and the availability of real-world big data, various mathematical methods for RI determination were developed, became more feasible and accurate and were compared in performance.^7^^,^^27^^,^^28^ Hoffmann, Bhattacharya, truncated minimum chi-square (TMC), and Kolmogorov-Smirnov distance (kosmic) indirect methods are the most widely used, and performed accordingly, in several studies.^7^^,^^28^^,^^29^

In the present study, regardless of the mean difference in insulin measurements, the Bhattacharya method was able to separate the Gaussian distributed “healthy” individuals for the determination of accurate indirect RI. This statement is validated by the similarity between DB1 and DB2 derived RI and the elevated diagnostic agreement rates for high FBI in all partitions. The lowest diagnostic agreement for elevated FBI found was 97.56%. These findings also indicate the accuracy of the indirect method used in this study.

To the best of our knowledge, this is a novel study to determine age and sex FBI RI through direct and indirect methods, for Brazilian adolescents, and to evaluate the indirect outcomes compared to the established standards of the direct method. These findings highlight the practicality, feasibility, cost-effectiveness, and accuracy of employing the Bhattacharya indirect method, facilitated by data mining of a large commercial LIS, in determining RI in adolescents. Significantly, this indirect method proves to be a reliable alternative, overcoming the inherent challenges associated with the conventional "gold standard" direct approach. Further studies using various indirect methods to determine RI in Brazilian pediatric populations should be encouraged, and further validations of the method in different analytes can be evaluated based on the large-scale ERICA study database. In the context of the prevailing big data era, Brazilian researchers are stimulated to contribute by presenting and publishing local RI through meticulous LIS data mining and indirect methodologies for local vulnerable populations, such as children and older adults.

## Funding

This research did not receive any funding from public, commercial, or not-for-profit organizations.

## Conflicts of interest

The authors declare no conflicts of interest.
